# Alterations in local chromatin environment are involved in silencing and activation of subtelomeric *var* genes in *Plasmodium falciparum*

**DOI:** 10.1111/j.1365-2958.2007.05899.x

**Published:** 2007-10

**Authors:** Till S Voss, Christopher J Tonkin, Allison J Marty, Jennifer K Thompson, Julie Healer, Brendan S Crabb, Alan F Cowman

**Affiliations:** 1 Division of Infection and Immunity, The Walter and Eliza Hall Institute of Medical Research Parkville 3050, Australia; 2 Department of Microbiology, Monash University Clayton 3800, Australia

## Abstract

*Plasmodium falciparum* erythrocyte membrane protein 1 (PfEMP1), encoded by the *var* gene family, undergoes antigenic variation and plays an important role in chronic infection and severe malaria. Only a single *var* gene is transcribed per parasite, and epigenetic control mechanisms are fundamental in this strategy of mutually exclusive transcription. We show that subtelomeric *upsB var* gene promoters carried on episomes are silenced by default, and that promoter activation is sufficient to silence all other family members. However, they are active by default when placed downstream of a second active *var* promoter, underscoring the significance of local chromatin environment and nuclear compartmentalization in *var* promoter regulation. Native chromatin covering the *SPE2*-repeat array in *upsB* promoters is resistant to nuclease digestion, and insertion of these regulatory elements into a heterologous promoter causes local alterations in nucleosomal organization and promoter repression. Our findings suggest a common logic underlying the transcriptional control of all *var* genes, and have important implications for our understanding of the epigenetic processes involved in the regulation of this major virulence gene family.

## Introduction

Phenotypic variation is a prerequisite for survival in a competitive and changing environment. In the case of pathogenic microorganisms, this is exemplified by the evolution of antigenic variation of cell surface proteins. Although various fundamentally different strategies for the regulation of antigenic variation have been described in different pathogens (Biggs *et al*., 1991; Deitsch *et al*., 1997; Pays *et al*., 2004; Al-Khedery and Allred, 2006; Dai *et al*., 2006), most rely on mutually exclusive expression of gene families; that is, only one family member is expressed in a single cell at any given time. By switching to the expression of a different antigenic variant, pathogens escape pre-existing immune responses, increasing the likelihood of chronic infection and successful transmission.

In *Plasmodium falciparum*, responsible for the most severe form of malaria in humans, erythrocyte membrane protein 1 (PfEMP1) undergoes such antigenic variation (Biggs *et al*., 1991; Roberts *et al*., 1992). PfEMP1, encoded by the 60-member *var* gene family (Baruch *et al*., 1995; Smith *et al*., 1995; Su *et al*., 1995), is exposed on the surface of infected erythrocytes, where it mediates cytoadherence to host endothelium and to uninfected erythrocytes (Baruch *et al*., 1996; Gardner *et al*., 1996; Rowe *et al*., 1997; Reeder *et al*., 1999). The resulting sequestration of red cell aggregates in the microvasculature of various organs is linked to severe complications, including cerebral and placental malaria (MacPherson *et al*., 1985; Carlson *et al*., 1990; Pongponratn *et al*., 1991; Berendt *et al*., 1994; Rowe *et al*., 1995; Beeson and Duffy, 2005). Hence, due to the combined phenotypes of antigenic variation and cytoadherence, PfEMP1 plays a crucial role in the chronicity of infection and malaria-associated morbidity and mortality.

*var* gene expression is controlled at the level of transcription and occurs in a mutually exclusive manner (Scherf *et al*., 1998). They are preceded by one of three major conserved upstream sequences (*upsA*, *upsB*, *upsC*), and a link exists between their chromosomal position and the upstream sequence they possess (Voss *et al*., 2000; Gardner *et al*., 2002). *var* genes in similar chromosomal locations were observed to share common directional orientations (Kraemer and Smith, 2003; Lavstsen *et al*., 2003). *UpsB var* genes are the most telomere-proximal genes in *P. falciparum* and are transcribed towards the centromere. *UpsA var* genes, and the *var2csa* gene flanked by the unique *upsE* sequence, are located next to and downstream of *upsB* genes and are transcribed towards the telomere. *UpsC var* genes exclusively occur in chromosome-internal clusters. These findings indicated a functional role of *var* upstream sequences in transcription, and indeed, *upsB* and *upsC* sequences were shown to possess weak promoter activity in transient transfection experiments (Deitsch *et al*., 1999; Voss *et al*., 2000). Additionally, sequence- and stage-specific DNA–protein interactions with possible functions in *var* gene repression and silencing were identified (Voss *et al*., 2003). The *var* intron plays an essential role in *upsC* silencing (Deitsch *et al*., 2001) and, although the underlying mechanisms remain obscure, this finding has been confirmed (Calderwood *et al*., 2003; Gannoun-Zaki *et al*., 2005; Dzikowski *et al*., 2006; Frank *et al*., 2006). Our recent work suggested that although *upsC* promoters are silenced by default in absence of the intron, silencing is enhanced in presence of this element (Voss *et al*., 2006). Recently, it has been shown that *var* gene transcription is controlled at the level of RNA polymerase II-dependent transcription initiation (Kyes *et al*., 2007).

An important role in global *var* gene regulation can be attributed to the intranuclear positioning of *var* genes. Most *var* genes are located in subtelomeric regions (Gardner *et al*., 2002) and, as in yeast, *P. falciparum* chromosome ends cluster at the nuclear periphery (Freitas-Junior *et al*., 2000). Chromosome-central *var* genes are also located at the nuclear periphery independent of their transcriptional state (Ralph *et al*., 2005; Marty *et al*., 2006; Voss *et al*., 2006). The perinuclear compartment is associated with enhanced transcriptional silencing in yeast and other eukaryotes (Andrulis *et al*., 1998; Cockell and Gasser, 1999; Gasser, 2001; Misteli, 2004). Likewise, in *P. falciparum* two subtelomeric genes, a chromosomal transgene and *var2csa*, were demonstrated to be reversibly silenced involving locus repositioning (Duraisingh *et al*., 2005; Ralph *et al*., 2005). Activation of artificial *var* loci occurs preferentially at chromosome-end clusters (Voss *et al*., 2006) and results in silencing of endogenous *var* gene transcription (Dzikowski *et al*., 2006; Voss *et al*., 2006). Together, these observations suggest the existence of a specialized perinuclear *var* transcription site.

Reversible histone modifications and ATP-dependent nucleosome remodelling provide the framework for the versatile epigenetic control of eukaryotic gene regulation (Jenuwein and Allis, 2001; Moazed, 2001; Rusche *et al*., 2003). In *P. falciparum*, *var* gene activation was shown to be associated with histone H4 acetylation (Freitas-Junior *et al*., 2005) and histone H3K9 trimethylation was linked to *var* gene silencing (Chookajorn *et al*., 2007). Additionally, a role for the *P. falciparum* histone deacetylase silent information regulator 2 (PfSIR2) in *var* gene silencing has been revealed (Duraisingh *et al*., 2005; Freitas-Junior *et al*., 2005). *P. falciparum* chromatin is organized into typical nucleosomal arrays even on transfected episomes (Horrocks *et al*., 2002), and activation of a silenced subtelomeric transgene was associated with different sensitivities of native chromatin to micrococcal nuclease (MNase) digestion (Duraisingh *et al*., 2005).

To date, functional investigation of *var* promoters has focused mainly on the chromosome-central *upsC* type (Deitsch *et al*., 2001; Calderwood *et al*., 2003; Gannoun-Zaki *et al*., 2005; Frank *et al*., 2006; Voss *et al*., 2006). Furthermore, while the above findings clearly highlight the important role of *var* gene regulatory sequences in reversible gene activation and mutual exclusion, the epigenetic processes and factors involved are poorly understood. Here, we investigated the role of subtelomeric *upsB* promoters in *var* gene regulation, and show that they are silenced by default and are functionally equivalent to chromosome-central *upsC* promoters. We demonstrate that two episomal *var* gene promoters *in cis* are activated simultaneously, implying that mutual exclusion is not based on single promoter competition but rather on locus activation. Low-resolution chromatin structure analysis identified two MNase-resistant regions in the *upsB* promoter, but revealed no difference in nucleosome positioning between opposite transcriptional states. Interestingly, one of the protected regions contains the previously identified regulatory *SPE2*-repeat array. Targeting of the *SPE2* binding activity to a heterologous promoter induces alterations in nucleosomal organization and promoter repression.

## Results

### Subtelomeric *upsB var* gene promoters are epigenetically regulated and silenced by default

**Fig. 1:**
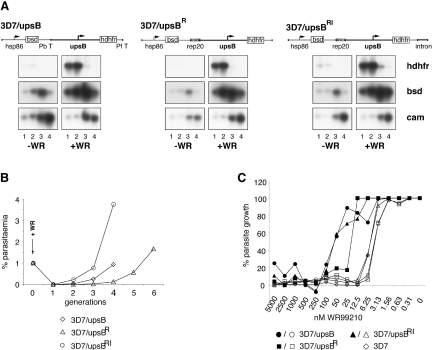
Epigenetic regulation of upsB *promoters.* A. Silencing and activation of *upsB*-regulated transcription. Northern blots showing episomal h*dhfr* and *bsd* transcription across the intra-erythrocytic asexual cycle in 3D7/upsB, 3D7/upsB^R^ and 3D7/upsB^RI^ before (–WR) and after (+WR) WR selection. Transcription of the endogenous *cam* gene serves as a stage-specific loading control. Vector maps are shown above each set of autoradiographs. *hsp86*, heat-shock protein 86 promoter; Pb T, *P. berghei* dhfr-thymidilate synthase terminator; *upsB*, *upsB* upstream sequence; Pf T, *P. falciparum hrp2* 3′ terminator; rep20, 0.5 kb rep20 repeats; intron, 0.6 kb *var* intron sequence. (1) 0–12 hpi (hours post-invasion); (2) 12–24 hpi; (3) 24–36 hpi; (4) 32–44 hpi. B. Growth assay. Blasticidin-S-selected parasites were challenged with WR at day 0. Parasite growth in the presence of WR was monitored over the following generations. Assays were repeated twice with the same result. C. WR sensitivities of 3D7/upsB, 3D7/upsB^R^ and 3D7/upsB^RI^ before (open) and after (filled) WR selection, and of 3D7 wild-type parasites.

In order to test whether *upsB* promoters play a role in the epigenetic regulation of subtelomeric *var* genes, we transfected constructs pHBupsB, pHBupsB^R^ and pHBupsB^RI^ containing two drug-resistance cassettes encoding (i) blasticidin deaminase (*bsd*), controlled by the *hsp86* promoter (for stable maintenance of episomes), and (ii) human dihydrofolate reductase (h*dhfr*), driven by an *upsB* promoter (Fig. 1A). A 0.5 kb sequence of rep20 repeats, which naturally occur upstream of subtelomeric *upsB* promoters and favour localization of episomes to perinuclear chromosome-end clusters (O'Donnell *et al*., 2002), was included in pHBupsB^R^ and pHBupsB^RI^. The *var* gene intron was added in pHBupsB^RI^ because its role in silencing has up to now only been demonstrated for *upsC* promoters (Deitsch *et al*., 2001; Calderwood *et al*., 2003; Gannoun-Zaki *et al*., 2005; Frank *et al*., 2006; Voss *et al*., 2006). After transfection, parasites were selected on blasticidin-S to generate 3D7/upsB, 3D7/upsB^R^ and 3D7/upsB^RI^. Northern blot experiments indicated that the *upsB* promoters were silenced by default in all three lines (Fig. 1A). All lines were also sensitive to challenge with WR consistent with the silenced h*dhfr* gene (Figs 1B and C). Continuous growth in the presence of WR, however, selected for WR-resistant populations after 3–6 generations. In these lines, h*dhfr* was transcribed at high levels in ring-stage parasites as a consequence of *upsB* promoter activation (Fig. 1A). The observation that episomal *upsB* promoters maintain the temporal activity profile of endogenous *upsB* promoters (Voss *et al*., 2003) indicates that the promoter region used is fully functional and contains the required regulatory elements. These data are consistent with what has previously been described for *upsC* promoters.

**Fig. 2:**
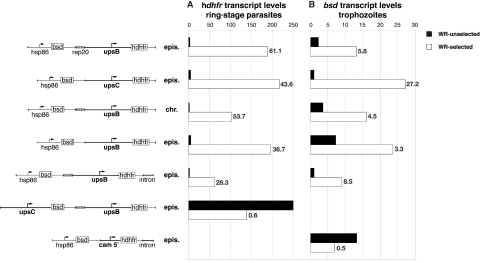
Analysis of *var* promoter activity by densitometry. A. Analysis of h*dhfr* transcript abundance in transfected 3D7 ring-stage parasites. Vector maps for each transfected line are shown on the left. The relative h*dhfr* transcript production per promoter before and after selection on WR is displayed (see also *Experimental procedures* and Fig. S1). B. Analysis of *bsd* transcript abundance in 3D7 trophozoites. The *cam* promoter construct at the bottom of the panel was used as control. The relative *bsd* transcript production per promoter before and after selection on WR is displayed. Numbers behind open bars (WR-selected) indicate the fold increase in steady-state transcripts compared with the default state (WR-unselected) (black bars). epis, episomal; chr, chromosomal.

To derive relative activity values for *upsB* promoters, we determined h*dhfr* transcript levels by densitometry (Figs 2 and S1). *UpsB* promoters produced 61-fold (pHBupsB^R^), 37-fold (pHBupsB) and 28-fold (pHBupsB^RI^) more transcripts after activation compared with the silenced state, similar to the level of *upsC* activation (44-fold). A comparable increase in activity (54-fold) was also measured after integration of pHBupsB^R^ into the subtelomeric *var* locus PFL0005w (Fig. S2). The silencing emanating from *upsB* promoters spread in *cis*, which was evident from the reduced *hsp86* promoter activity on silenced episomes (Fig. 2). While the intron is not required for *upsB* silencing, it appears to decrease *upsB* activity (Fig. 2), a tendency we also observed with *upsC* promoters (Voss *et al*., 2006). In contrast to *upsC* promoters, however, *upsB* promoter activation did not occur less frequently in the presence of the intron as demonstrated by the similar sensitivities of 3D7/upsB, 3D7/upsB^R^ and 3D7/upsB^RI^ parasites to WR challenge (Fig. 1B), suggesting that the *var* intron does not augment silencing of *upsB* promoters.

### *UpsB* promoter activation interferes with mutually exclusive *var* expression

**Fig. 3:**
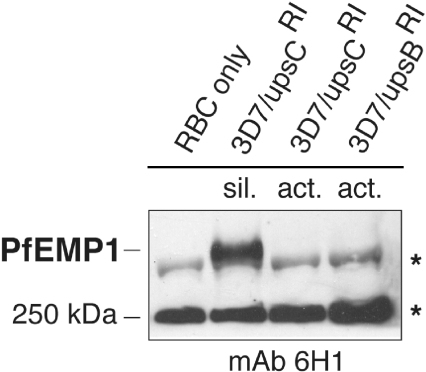
PfEMP1 is not expressed in *var* knock-down parasites. Detection of PfEMP1 by Western analysis of Triton X-100-insoluble/SDS-soluble membrane fractions from uninfected (control) and iRBCs. The two bands at approximately 250 kDa (asterisks) represent cross-reactive α- and β-spectrin (Cooke *et al*., 2006). sil., silenced *var* promoter; act., activated *var* promoter.

Our findings reveal striking similarities between the regulation of subtelomeric *upsB* and chromosome-central *upsC* promoters, suggesting that silencing of all *var* genes is generally induced by their *cis*-linked regulatory elements, independent of chromosomal location and/or promoter type. Moreover, activation of artificial *var* loci, both episomal and chromosomal, prevents transcription of endogenous *var* genes, emphasizing the central role of *var* promoters in mutually exclusive *var* gene transcription (Dzikowski *et al*., 2006; Voss *et al*., 2006). However, for *upsB* promoters this effect was only demonstrated for a chromosomal locus (Dzikowski *et al*., 2006). To test whether activation of artificial *upsB* loci carried on episomes also interferes with mutual exclusion, we investigated the ability of WR-selected 3D7/upsB^RI^ parasites to express PfEMP1 and to adhere to the endothelial receptor CD36. Similar to WR-selected 3D7/upsC^RI^ parasites (Voss *et al*., 2006), we find that 3D7/upsB^RI^ expressing the h*dhfr*-resistance gene failed to express PfEMP1 (Fig. 3). Consequently, adherence to CD36 of red blood cells (RBCs) infected with WR-selected 3D7/upsB^RI^ occurred at an average of 14.4% (13.8% and 15%) compared with unselected parasites. Moreover, fluorescent *in situ* hybridization (FISH) revealed a significant difference (*P*< 0.05) in the colocalization of pHBupsB with telomeric clusters in WR-unselected (39 ± 2.8%; mean *n* = 113) and WR-selected 3D7/upsB parasites (62 ± 3.5%; mean *n* = 102). This finding indicates that *upsB* promoter activation occurs in perinuclear chromosome-end clusters and provides a further parallel to the regulation of *upsC* (Voss *et al*., 2006) and *upsE* (Marty *et al*., 2006) promoters.

### Two episomal *var* promoters *in cis* are simultaneously active

**Fig. 4:**
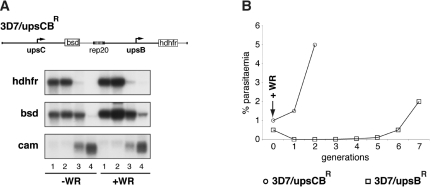
Simultaneous activation of two *var* gene promoters *in cis*. A. Activity of the *upsC* and *upsB* promoter in 3D7/upsCB^R^ parasites before (–WR) and after (+WR) WR selection. Northern blots showing episomal h*dhfr* and *bsd* transcription across the intra-erythrocytic cycle. Transcription of the endogenous *cam* gene serves as a stage-specific loading control. The vector map is shown on top. (1) 0–12 hpi; (2) 12–24 hpi; (3) 24–36 hpi; (4) 32–44 hpi. B. Growth assay. Blasticidin-S-selected 3D7/upsCB^R^ and 3D7/upsB^R^ parasites were challenged with WR at day 0. Parasite growth in the presence of WR was monitored over the following generations.

Because *P. falciparum* episomes are maintained as concatamers of tandemly repeated plasmids (O'Donnell *et al*., 2001), it was impossible in previous studies to determine whether only a single episomal *var* promoter was activated at any time, or whether instead multiple *var* promoters were active simultaneously on the same DNA molecule. To test this, we designed construct pHBupsCB^R^, where two *var* gene promoters, *upsC* and *upsB*, control transcription of the *bsd* and h*dhfr* reporter genes respectively (Fig. 4A). Transfection of pHBupsCB^R^ followed by selection on blasticidin-S selected for the transgenic line 3D7/upsCB^R^ Northern analysis revealed that *upsC* promoted transcription of the *bsd* gene in ring-stage and early trophozoite parasites as expected. Strikingly, in this context the downstream *upsB* promoter was fully activated by default, demonstrating that local *var* promoter activation dominates over silencing (Figs 2 and 4A). Consistent with this finding, WR-unselected 3D7/upsCB^R^ parasites were resistant to WR challenge unlike 3D7/upsB^R^ parasites, where the *upsB*-h*dhfr* cassette was silenced (Fig. 1A and 4B). Together, these results prove that multiple episomal *var* promoters *in cis* are active simultaneously. We hypothesize that this is related to the absence of boundary/insulator elements on the plasmids, which are naturally required to prevent simultaneous activation of neighbouring *cis*-linked *var* genes. These findings furthermore suggest that the local chromatin environment is important to determine the transcriptional state of *var* gene promoters.

### Evidence for positioned nucleosomes in *var* gene promoters

**Fig. 5:**
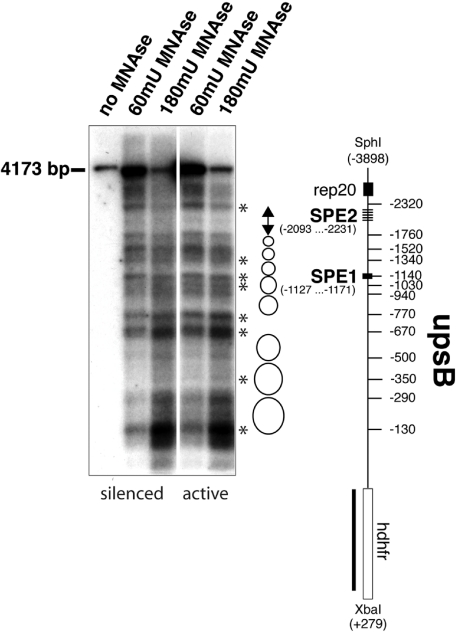
Low-resolution chromatin analysis of episomal *upsB* promoters. Native chromatin was digested with MNase followed by indirect end-labelling. Southern blots were hybridized with a 257 bp h*dhfr* probe to detect MNase-sensitive sites in the silenced and activated *upsB* promoters on pHBupsB^R^. A map of the 4172 bp XbaI/SphI fragment of pHBupsB^R^ containing the *upsB* promoter is shown on the right. MNase-sensitive sites are highlighted with respect to the h*dhfr* start codon. The positions of the *cis*-acting elements *SPE1* and *SPE2* and the rep20 repeat region are indicated. The arrow depicts the MNase-resistant region (−1760 to −2320) containing five direct *SPE2* repeats (−2093 to −2231) (Voss et al., 2003). Asterisks identify sites that are also preferentially cut in naked plasmid DNA (Fig. S3). Circles represent a proposed nucleosomal organization.

We recently reported differences in local chromatin structure between the silenced and active states of a subtelomeric transgene in *P. falciparum* (Duraisingh *et al*., 2005). Here we have investigated whether such alterations are also important in the regulation of *var* gene promoters. Indirect end-labelling of native chromatin in 3D7/upsB^R^ parasites revealed a pattern of MNase-sensitive sites, which might be indicative for a number of positioned nucleosomes (Fig. 5). A number of MNase-sensitive sites were also preferentially cut in naked plasmid DNA (Fig. S3), and due to the low-resolution mapping strategy employed here, it remains unknown whether these sites are truly positioned between adjacent nucleosomes. Two highly accessible sites are located within the region containing the transcriptional start site (−515 to −804) (Voss *et al*., 2003), followed by a protected area within the 5′ untranslated region. A 200 bp region in the promoter (−940 to −1140) contains three consecutive MNase-sensitive sites, positioned directly downstream of the *SPE1* regulatory element (−1127 to −1171) (Voss *et al*., 2003). A 500 bp region upstream (−1760 to −2320) was highly resistant to digestion even at high enzyme concentrations. This region contains the regulatory *SPE2*-repeat array (−2093 to −2231) (Voss *et al*., 2003), suggesting that the *SPE2* binding activity may play a key role in chromatin organization and epigenetic regulation. However, we neither observed significant differences between the silenced and active states of *upsB* (Fig. 5) or *upsC* promoters (data not shown), nor did we detect any effects conferred by rep20 repeats or the *var* intron on the local chromatin structure of *var* gene promoters (data not shown).

### Regulatory *SPE2* elements induce local chromatin compaction and repress the activity of a heterologous promoter

**Fig. 6:**
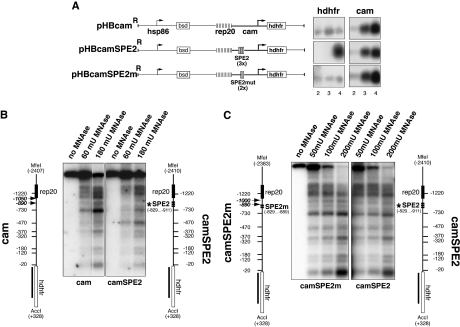
Insertion of *SPE2* elements into the heterologous *cam* promoter. A. The effect of *SPE2* insertion on *cam* promoter timing. Plasmid maps are shown on the left. Stage-specific Northern blots to detect h*dhfr* and *cam* (control) transcription is shown on the right. (2) 12–24 hpi; (3) 24–36 hpi; (4) 32–44 hpi. B. Comparison of the chromatin structure of the episomal *cam* and *camSPE2* promoters. MNase-sensitive sites are indicated with respect to the h*dhfr* start codon. The position of the inserted *SPE2* elements (−829 to −911) in *camSPE2* is shown. *SPE2* insertion causes local protection from MNAse digestion (asterisk), and the two MNAse-sensitive sites in the *cam* wild-type promoter disappear (arrows). C. Comparison of the chromatin structure of the episomal *camSPE2* and *camSPE2m* promoters. The positions of the inserted *SPE2* (−829 to −911) and *SPE2m* (−829 to −889) elements are shown. Note that the two MNase-sensitive sites in the *cam* wild-type promoter (−890 and −1050) are retained after insertion of the mutated *SPE2m*.

We were interested in testing the potential role of *SPE2* elements in chromatin organization and transcriptional regulation. We generated construct pHBcamSPE2^R^ with three tandem copies of *SPE2* inserted into the heterologous *P. falciparum* calmodulin (*cam*) promoter (Fig. 6A). pHBcam^R^ (Voss *et al*., 2006) and pHBcamSPE2m^R^, where point mutations in the *SPE2* sequence prevent the interaction with the *SPE2* binding protein (Voss *et al*., 2003), were used as controls. The three blasticidin-S-resistant transgenic lines 3D7/cam, 3D7/camSPE2^R^ and 3D7/camSPE2m^R^ were resistant to WR challenge, indicating that the *cam* promoter was not silenced by insertion of *SPE2* elements (data not shown). However, insertion of *SPE2* strongly affected the temporal activity profile of the *cam* promoter. The activity of the *camSPE2* promoter was restricted to schizont-stage parasites [32–44 h post invasion (hpi)]. In contrast, the wild-type *cam* promoter was active across much of the intra-erythrocytic cycle, and insertion of mutated *SPE2* elements had no effect (Fig. 6A). To obtain evidence that this observation was due to the actual interaction of the *SPE2* binding activity with its cognate binding site, we analysed the chromatin structure in these promoters by indirect end-labelling. The pattern of MNase-sensitive sites in the episomal wild-type *cam* promoter was similar to the pattern observed in a *cam* promoter at a subtelomeric transgene locus (Duraisingh *et al*., 2005). However, after insertion of the *SPE2* elements at position −833, the two sensitive sites at −890 and −1050 were now protected from MNase digestion (Fig. 6B). Furthermore, these alterations were specific to the *SPE2* elements as insertion of the mutated *SPE2* repeats had no effect (Fig. 6C). These findings are consistent with the MNase resistance of the *SPE2* array in *upsB* promoters, and indicate that the change in temporal *cam* promoter activity is a direct result of *SPE2* binding factor recruitment to the introduced *SPE2* elements.

## Discussion

*var* gene promoters are of central importance in epigenetic regulation and mutual exclusion of *var* gene transcription. Previous studies focused almost exclusively on the role of *upsC* promoters controlling the expression of chromosome-central *var* genes. Here we show that subtelomeric *upsB* promoters (associated with the largest *var* gene subgroup) display similar functional properties: (1) *upsB* promoters are regulated by epigenetic mechanisms to establish either a silenced (default) or active state; (2) the transcriptional state is inherited over many generations in the absence of selective pressure; (3) *upsB* silencing and activation can spread *in cis,* affecting neighbouring loci; and (4) activation of episomal *upsB* promoters occurs preferentially at chromosome-end clusters at the nuclear periphery and interferes with mutual *var* gene exclusion, preventing expression of PfEMP1.

Several studies investigating *var* promoter function in transiently or stably transfected parasites suggested that *upsC* promoters are activated by default and only silenced in the presence of a *cis*-linked *var* intron (Deitsch *et al*., 2001; Gannoun-Zaki *et al*., 2005; Frank *et al*., 2006). In contrast, in this study we have demonstrated efficient silencing of *upsC* (Voss *et al*., 2006) and *upsB* promoters in absence of the intron. Our results are consistent, however, with a role of the *var* intron in insulator/boundary function and in controlling the degree of promoter activation particularly for *upsC*. An interesting explanation for this ‘intron controversy’ was recently proposed (Frank and Deitsch, 2006) and is related to the intron's own promoter activity (Calderwood *et al*., 2003). Heterologous promoters (including the *hsp86* promoter) were able to functionally replace the role of the intron in *upsC* silencing (Frank and Deitsch, 2006). It was proposed that a *cis*-linked promoter activity, rather than the intron sequence itself, mediates cooperative *var* promoter silencing. This would suggest that the *hsp86* promoter present on all constructs in this and our previous study (Voss *et al*., 2006) may have functionally replaced the intron to silence *var* promoters. However, Viebig *et al*. (2005) found that integration into the *var2csa* locus of a h*dhfr*-resistance cassette under control of the calmodulin promoter led to the constitutive activation, rather than silencing, of *upsE*-driven transcription of the truncated *var2csa* gene. Hence, and in our view more likely, differences in transfection vector design and/or the drug selection strategy applied to select for transfected parasites (h*dhfr* versus *bsd*) may explain these contradictory results. Whatever the reason, the exact role of the intron in *var* promoter silencing remains elusive and cannot be determined from the existing information, and other approaches are clearly required to clarify this issue.

*var* genes are located at the nuclear periphery independent of their transcriptional state, and switches in *var* transcription are linked to perinuclear locus repositioning (Duraisingh *et al*., 2005; Ralph *et al*., 2005; Voss *et al*., 2006). A current model suggests a unique perinuclear domain permissive for transcription of a single *var* locus. Consistent with this hypothesis, drug-induced activation of episomal *var* promoters is sufficient to compete with endogenous *var* transcription (Dzikowski *et al*., 2006; Voss *et al*., 2006). However, due to the concatameric structure of episomes in *P. falciparum*, previous studies failed to reveal whether the basis for mutual exclusion is truly related to direct *var* promoter competition. To address this issue, we designed plasmid pHBupsCB^R^ carrying two *cis*-linked *var* promoter cassettes. In presence of an activated upstream *upsC* promoter, the *upsB* promoter was fully activated by default. This is in striking contrast to pHBupsB^R^, where the *upsB* promoter is naturally silenced. Therefore, in a closely *cis*-linked context, two *var* promoters can be active simultaneously and *var* promoter activation is dominant over silencing. This suggests that mutual *var* exclusion involves activation of a chromosomal *var* locus through chromatin alterations transforming the regulatory regions into a transcription-competent state. Co-activation of neighbouring chromosomal *var* genes in their native context is presumably prevented by boundary elements that sequester the spread of active chromatin. While this scenario is consistent with the existence of a perinuclear domain competent for *var* gene transcription, it also highlights that a model in which such a domain would be physically restricted to activate only a single *var* gene is not sufficient to explain mutually exclusive *var* transcription.

Despite the fact that all *var* genes are wired into the same allelic exclusion programme, it is noteworthy that different epigenetic factors appear to be involved in the regulation of different *var* gene subsets. As demonstrated by microarray analysis, the *var2csa* and *upsA var* genes were de-silenced in PfSIR2 knock-out parasites, whereas transcription of *upsB* and *upsC var* genes was largely unaffected (Duraisingh *et al*., 2005). ChIP experiments revealed the presence of PfSIR2 at the silenced *var2csa* gene but not a chromosome-central *upsC* locus (Freitas-Junior *et al*., 2005). However, when activated, both genes were associated with acetylated histone H4, indicating the involvement of multiple histone deacetylases in *var* gene silencing. Furthermore, recent data have identified an epigenetic mark (H3K9 trimethylation) linked to silencing and transcriptional memory of *upsB* and *upsC var* genes (Chookajorn *et al*., 2007). Together, this is additional evidence for the evolution of the *var* gene family into functional and commonly regulated subsets that might play distinct roles in parasite biology and malaria pathogenesis.

Apart from differential histone modifications, silenced and active states of epigenetically regulated genes are often associated with alterations in nucleosomal organization. In general, silenced genes in *Saccharomyces cerevisiae* heterochromatic regions display an ordered array of regularly spaced nucleosomes and decreased sensitivity to various endonucleases (Richards and Elgin, 2002; Rusche *et al*., 2003). Similarly, we showed previously that a transgene inserted into subtelomeric heterochromatin in *P. falciparum* displayed altered sensitivity to MNase digestion, suggesting the participation of ATP-dependant nucleosome remodelling enzymes in the establishment of the silenced and active states. Our analysis of the chromatin structure in *var* gene promoters did not detect significant differences between opposite transcriptional states. Furthermore, silenced and active *upsC* chromatin displayed an identical MNase-sensitivity pattern in both the absence and presence of a downstream intron (data not shown). It is possible that opposing transcriptional states of *var* promoters are indeed associated with alterations in translational or rotational nucleosome positioning but remained undetected by the low-resolution mapping approach used here, or that altered susceptibility to endonucleases other than Mnase, such as DNase I or restriction enzymes, exists. However, lack of altered chromatin sensitivity to endonucleases was also observed in Polycomb group-dependent silencing in *Drosophila melanogaster* (Pirrotta and Gross, 2005) and in nuclear hormone receptor-mediated gene regulation (Urnov and Wolffe, 2001). Moreover, many studies in yeast compared the chromatin structure of loci silenced in wild-type cells and activated in mutant strains lacking essential heterochromatin components.

The pattern of MNase-sensitive sites is consistent with a mainly nucleosomal organization of *upsB* chromatin and a number of positioned nucleosomes. The sensitive site at −1140 approximates the position of the regulatory *SPE1* element, indicating that this motif may be located and accessible between two adjacent nucleosomes. Most noteworthy, however, is the highly resistant region between positions −1760 and −2320 containing five tandem repeats of the *SPE2* motif. The *SPE2* binding factor(s) bind cooperatively and with high avidity to their cognate binding sites (Voss *et al*., 2003), suggesting a role in chromatin organization. The effects achieved by insertion of *SPE2* into the context of the *cam* promoter clearly support this hypothesis: introduction of *SPE2* leads to a local reorganization and compaction of chromatin. As a result, the modified *cam* promoter was rendered completely repressed throughout most of the intraerythrocytic cycle and was only active during the schizont stage. The fact that soluble *SPE2* binding activities are absent in nuclear extracts from ring- to early schizont-stage parasites (Voss *et al*., 2003) indicates that the entire pool of this factor is tightly associated with their target sites. We therefore speculate that targeted recruitment of the *SPE2* binding activity to the *cam* promoter causes repression/silencing until the intra-erythrocytic replication cycle. During this stage, a window opens where repeated replication cycles provide newly synthesized expression cassettes that can be active in absence of soluble *SPE2* binding protein. Following expression of the *SPE2* binding activity late in the cycle, repressive complexes are re-established. However, it appears that the *SPE2* binding factors remain associated with *upsB* promoters even after activation (Fig. 5) and *upsB* promoters are still silenced after removal of the *SPE2* repeats (Fig. S1). Together this suggests that while the role of this interaction in its native context may involve the establishment and maintenance of *upsB* silencing, it is neither sufficient for *upsB* silencing, nor is its inhibition necessary for activation.

In summary, our findings suggest that, despite the marked differences in *upsC* and *upsB* promoter sequence and the absence of detectable common regulatory motifs, the regulation of the entire *var* gene repertoire obeys the same overall logic. We hypothesize that each *var* gene locus represents an individual transcription unit equipped with functional promoter and intron elements. Regional chromatin modifications are initiated at *cis*-acting regulatory motifs and progress locally to mediate both *var* gene silencing and activation.

## Experimental procedures

### Parasite cultures, transfection and drug response assays

*Plasmodium falciparum* 3D7 parasites were cultured as described previously (Trager and Jenson, 1978). Growth synchronization was achieved by repeated sorbitol lysis (Lambros and Vanderberg, 1979). Transfection and drug sensitivity assays were performed as described (Voss *et al*., 2006).

### Transfection constructs

pHBupsB^R^ was generated by replacement of the BglII/BamHI *cam* promoter in pHBcam^R^ (Voss *et al*., 2006) with the 2621 bp *upsB* promoter amplified from pCAT4A3 (Voss *et al*., 2000). pHBupsB^RI^ was derived in the same way from pHBcam^RI^. pHBupsB lacks the 0.5 kb rep20 repeat sequence in pHBupsB^R^ and was generated by digestion of pHBupsB^R^ with PstI/XcmI, deleting the rep20 repeat region including 140 bp of the 5′ end of the *upsB* promoter up to the XcmI site (−2481), followed by ligation of a 140 bp PstI/XcmI PCR fragment amplified from pCAT4A3 to restore the full-length *upsB* promoter. The *hsp86* promoter in pHBupsB^R^ was excised with KpnI and BstBI and replaced with the full-length *upsC* promoter to generate pHBupsCB^R^. *SPE2 var* gene promoter elements, and mutated *SPE2* elements (*SPE2m*) (Voss *et al*., 2003) were cloned as blunt-ended double-stranded oligonucleotides into the EcoRV site (−831 bp) of the *cam* promoter in pLT-3 (A. Maier, unpublished). Modified *cam* promoters were amplified from, and used to replace, the wild-type *cam* promoter in pHBcam^R^ to generate pHBcamSPE2^R^ and pHBcamSPE2m^R^. pHBcamSPE2^R^ carries three wild-type head-to-tail copies of *SPE2*, and pHBcamSPE2m^R^ carries two mutated copies of *SPE2* (*SPE2m*).

### Northern analysis

Total RNA was isolated at four time points across the 48 h intra-erythrocytic cycle (time point 1, 0–12 hpi; time point 2, 12–24 hpi; time point 3, 24–36 hpi; time point 4, 32–44 hpi) from parasites synchronized three times. RNA isolated from equal numbers of nuclei for each sample was loaded (time points 1 and 2, 10^7^ parasites; time point 3, 0.75 × 10^7^ parasites; time point 4, 0.25 × 10^7^ parasites). Northern blots were hybridized with [α-^32^P]dATP-labelled probes detecting h*dhfr*, *bsd*, *cam*, *msp8* and *hsp86* transcription (Figs 1, 4 and S1). Densitometry was used to quantify transcript levels (Fig. S1). To account for differences in RNA loading, the abundance of h*dhfr* transcripts was adjusted by comparison with the endogenous ring stage-specific *msp8* transcription. *bsd* transcription in trophozoites was adjusted by comparison with endogenous *hsp86* transcription. Values were further normalized for plasmid copy numbers (Fig. S1). Thus, the results shown in Fig. 2 represent relative amounts of transcript produced by a single promoter in each transfected line before and after selection on WR. Figures 1, 4 and S1 show results obtained from single blots each.

### Southern analysis

For plasmid copy number determination, gDNA was digested with EcoRV/PvuII. Copy number was determined by densitometry after hybridization with the [α-^32^P]dATP-labelled *hrp2* 3′UTR probe comparing the signal intensities derived from the endogenous single-copy *pfhrp2* locus (8317 bp) with the plasmid-encoded *hrp2* 3′UTR sequences. Integration of episomes into chromosomal DNA was mapped by hybridization with the h*dhfr* probe and an 846 bp fragment derived from the 5′ end of PFL0005w (+544 to +1390) to HindIII/XhoI-digested gDNA.

### Cytoadherence of infected RBCs (iRBCs), Western analysis and FISH

Cytoadherence of parasitized erythrocytes to CD36 was analysed as described (Beeson et al., 1999). RBC pellets were used to extract PfEMP1 into the Triton X-100-insoluble, SDS-soluble fraction as described (van Schravendijk et al., 1993). Aliquots (5 × 10^6^ RBCs, 4% trophozoites) were resolved on a reducing 5% polyacrylamide gel and Western blots probed with mouse monoclonal anti-PfEMP1 antibody 6H1 diluted 1:200 (Duffy et al., 2002). FISH analysis was performed using ring-stage parasites as presented elsewhere (Voss et al., 2006), using telomere-associated repeat element 4 and pGEM probes for the detection of chromosome-end clusters and the plasmid backbone respectively.

### Indirect end-labelling

Parasites transfected with *var* promoter constructs were analysed at the ring stage (approximately 7.5 ml packed iRBCs, 5% parsitaemia). Parasites transfected with *cam* promoter constructs were harvested at the trophozoite/schizont stage (1.5 ml packed iRBCs, 5% parasitaemia). Parasites were released by saponin lysis of iRBCs, washed twice in ice-cold PBS and permeabilized in 1 ml of ice-cold chromatin digestion buffer [20 mM Tris-HCl (pH 7.5, 15 mM KCl, 60 mM NaCl, 1 mM CaCl_2_, 5 mM MgCl_2_, 300 mM sucrose, 0.4% NP-40] for 5 min on ice. F200 μl aliquots were equilibrated at 37°C for 2 min and digested for 3 min at 37°C by adding 5 μl of MNase dilutions (MBI Fermentas) at 10–80 mU μl^−1^. Reactions were stopped with 40 μl of stop buffer (100 mM EDTA, 4% SDS) and 66 μl of 2.5 M NaCl. Samples were treated with 100 μg proteinase K and 50 μg RNase A for 2–4 h at 50°C. Digested chromatin was extracted twice with phenol/chloroform, precipitated with 2.5 vols 100% ethanol and resuspended in 30 μl TE buffer [10 mM Tris-HCl (pH 8.0), 1 mM EDTA]. Control digests using purified plasmid DNA were performed as above. For indirect end-labelling, samples were digested to completion with restriction enzymes, resolved on 1–1.4% agarose gels, blotted onto Hybond-XL membranes (Amersham) and probed with a [α-^32^P]dATP-labelled h*dfhr* probe.

## Supplementary material

This material is available as part of the online article from: http://www.blackwell-synergy.com/doi/abs/10.1111/j.1365-2958.2007.05899.x (This link will take you to the article abstract).
