# Supplementary material for: Alterations in local chromatin environment are involved in silencing and activation of subtelomeric var genes in Plasmodium falciparum
Source: Mol Microbiol. 2007 Oct;66(1):139–50. doi: 10.1111/j.1365-2958.2007.05899.x (PMC2169929; doi:10.1111/j.1365-2958.2007.05899.x)
Supplement: Supplementary file 1 [file mmi0066-0139-SD1.pdf]

Supplementary Figure 1
Voss et al.

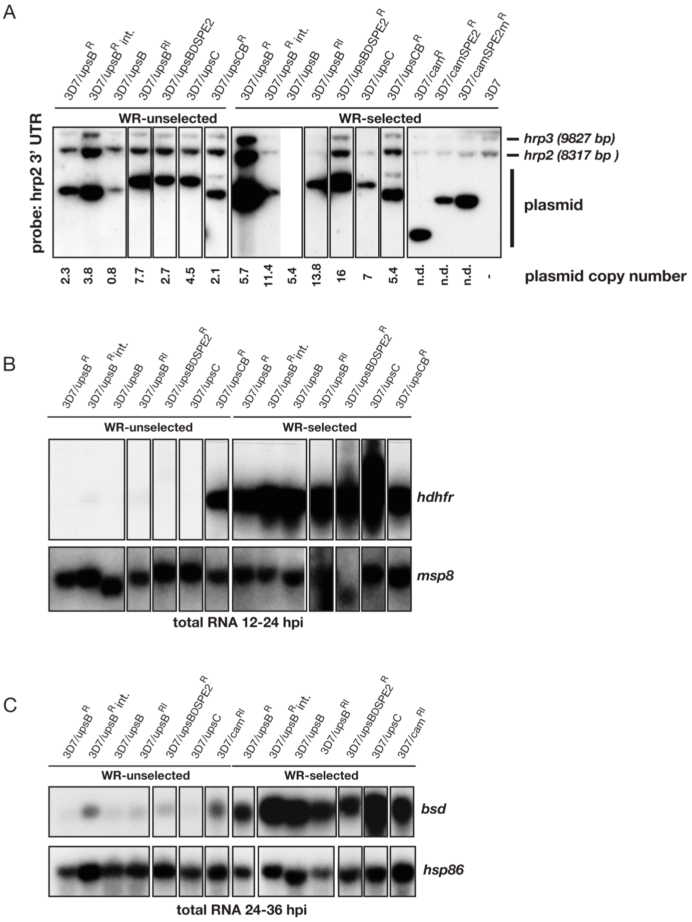

Supplementary Figure 1. Densitometrical analysis of Southern and Northern blot signals.

A. Southern blot probed with a *hrp2* terminator probe to simultaneously detect transfected plasmids and the endogenous single-copy control loci *hrp2* and *hrp3* (cross-reactive with the *hrp2* probe). Sizes of the *hrp2* and *hrp3* fragments are shown on the left. Plasmid copy numbers in individual lines are shown at the bottom and were determined densitometrically by calculating the ratio between plasmid and *hrp2* signal intensities. Various autoradiograph exposure times were analysed and signal intensities measured in the linear range for each transfected line. All samples were analysed on the same blot.

B. Northern analysis of h*dhfr* transcription in ring stage parasites (12-24 hpi). Transcription of the *msp8* gene was used as a control for RNA loading and ring stage specificity. Various autoradiograph exposure times were analysed and signal intensities measured in the linear range for each transfected line. Relative h*dhfr* transcript levels were determined by adjusting the h*dhfr* signal intensities for loading differences (*msp8* signal) and plasmid copy numbers.

(C) Northern analysis of *hsp86* promoter-driven *bsd* transcription in trophozoites (24-32 hpi). Transcription of the endogenous *hsp86* gene was used as a control. Various autoradiograph exposure times were analysed and signal intensities measured in the linear range for each transfected line. Relative *bsd* transcript levels were determined by adjusting the *bsd* signal intensities for loading differences (*hsp86* signal) and plasmid copy numbers.

Supplementary Figure 2
Voss et al.

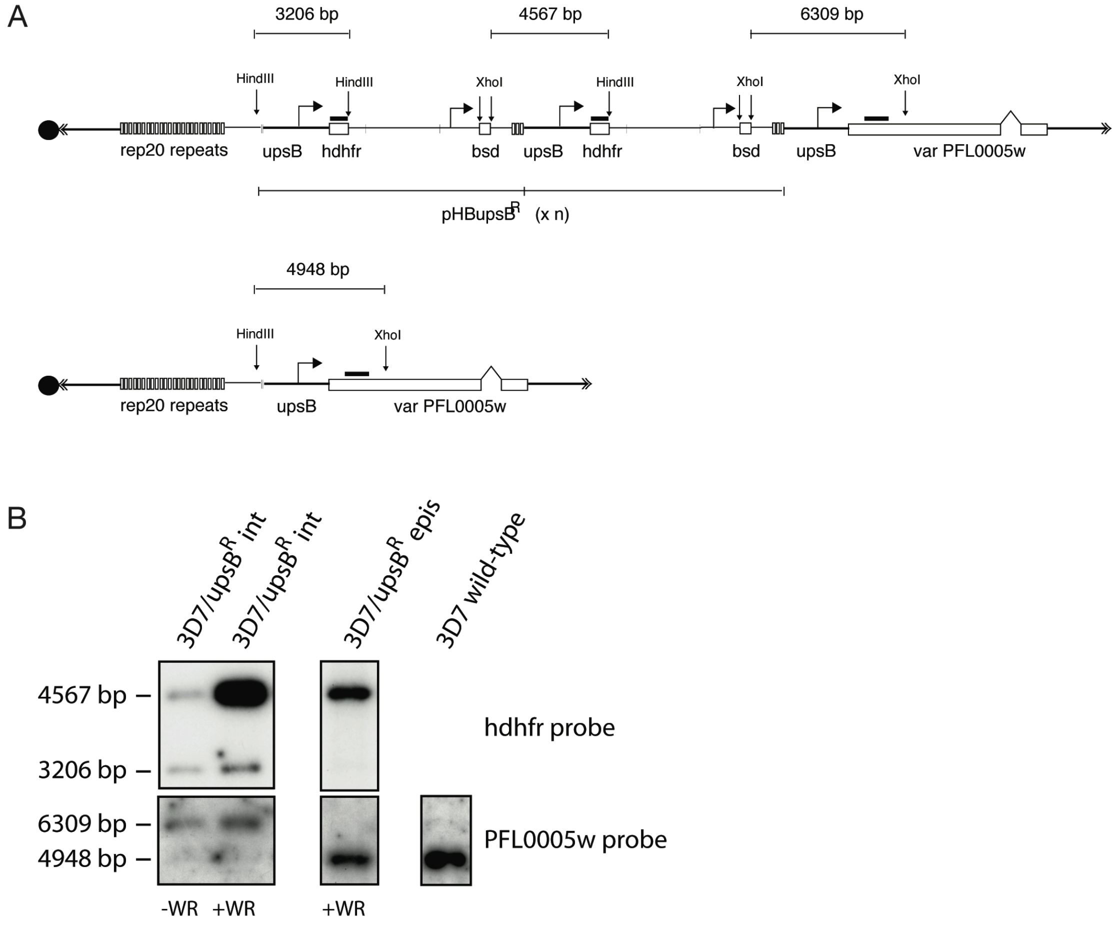

Supplementary Figure 2. Integration of pHBupsB^R into the subtelomeric upsB locus PFL0005w.

A. Maps of the integration event and the wild-type PFL0005w locus are shown on top. *Hind*III and *Xho*I restriction sites used for Southern analysis and fragment lengths are indicated. Probes used to detect the h*dhfr* gene and PFL0005w are indicated by black bars.

B. Southern analysis demonstrating the integrated and episomal forms of pHBupsB^R in 3D7/upsB^R parasites.

Supplementary Figure 3
Voss et al.

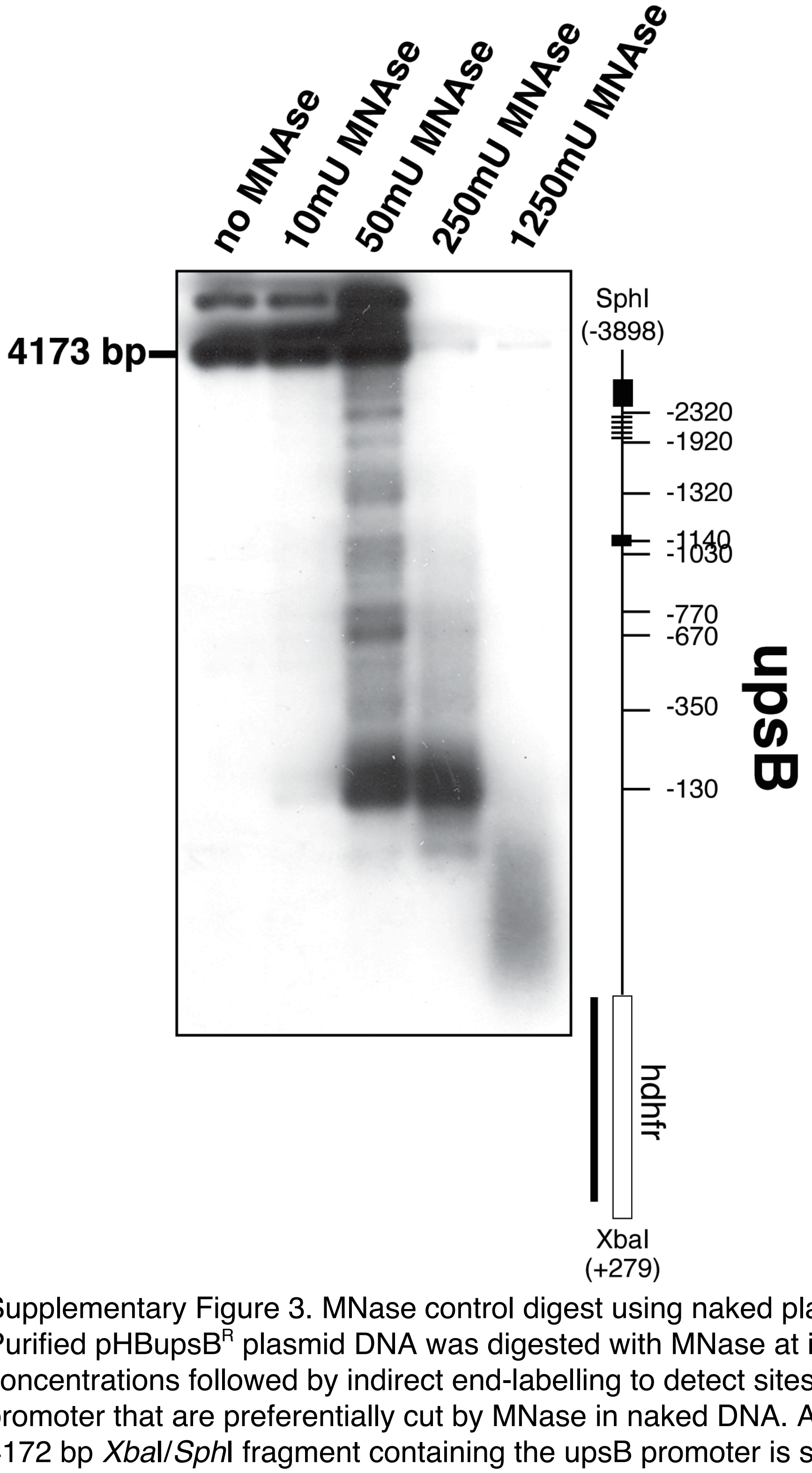

Supplementary Figure 3. MNase control digest using naked plasmid DNA. Purified pHBupsB^R plasmid DNA was digested with MNase at increasing concentrations followed by indirect end-labelling to detect sites in the upsB promoter that are preferentially cut by MNase in naked DNA. A map of the 4172 bp *Xba*I/*Sph*I fragment containing the upsB promoter is shown on the right. MNAse-sensitive sites are highlighted with respect to the h*dhfr* start codon.
